# Protein language model-generated enzyme sequences exhibit high stability in molecular dynamics simulations

**DOI:** 10.3389/frai.2026.1844189

**Published:** 2026-08-31

**Authors:** Enrico Mario Alessandro Fassi, Marco Nicolini, Emanuele Saitto, Giorgio Valentini, Elena Casiraghi, Giovanni Grazioso

**Affiliations:** 1 Dipartimento di Scienze Farmaceutiche, Università degli Studi di Milano, Milan, Italy; 2 AnacletoLab – Dipartimento di Informatica, Università degli Studi di Milano, Milan, Italy; 3 ELLIS – European Laboratory for Learning and Intelligent Systems, Milan, Italy

**Keywords:** conditional transfer learning, enzyme sequence generation, molecular dynamics, protein language models, transformers

## Abstract

**Introduction:**

Large Language Models (LLMs) have transformed protein engineering by capturing complex sequence patterns from large datasets, enabling applications such as structure prediction and functional annotation. Finenzyme applies conditional transfer learning to generate biologically plausible enzyme sequences conditioned on Enzyme Commission (EC) numbers.

**Methods:**

In this work, we extended Finenzyme with an in silico selection pipeline that first identifies generated sequences most likely to preserve or enhance the functional characteristics of specific EC categories and then evaluates them through molecular dynamics (MD) simulations to assess their structural stability and conformational dynamics.

**Results:**

MD simulations of 236 Finenzyme-generated enzymes across four EC classes (59 *µ*s total simulation time) confirmed high structural stability. Across all enzyme classes, 74-95% of the models maintained stable tertiary structures and correct folding throughout the trajectories, with 195 out of 236 structures (82.6%) exhibiting sustained stability.

**Discussion:**

By combining conditional pre-trained language model fine-tuning with dynamic structural evaluation, our framework advances beyond static sequence-based predictions to address the structural and functional dimensions of enzyme behavior, key aspects for both biomedical and industrial applications.

## 1 Introduction

**Figure 1:**
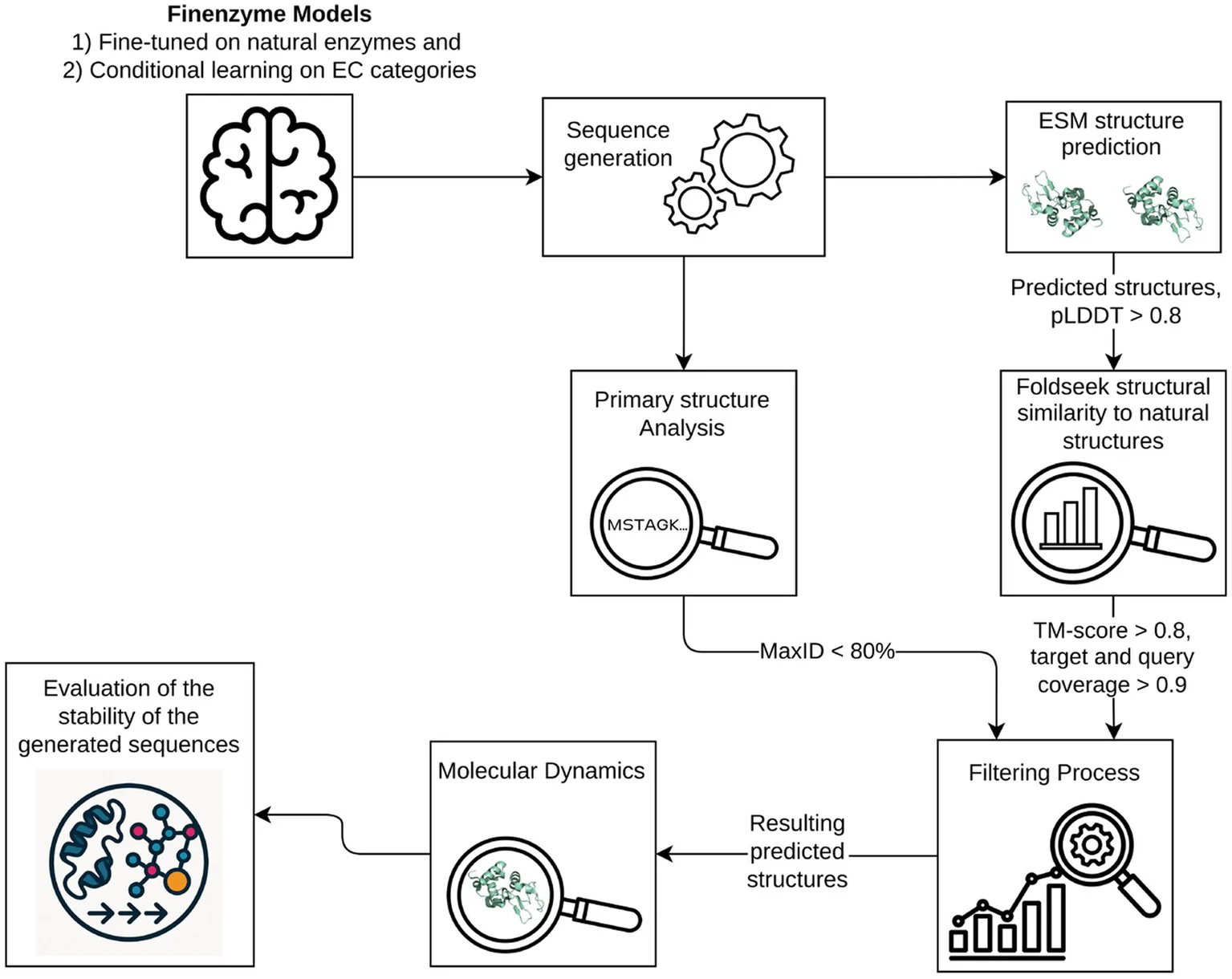
Schema of the performed work.

Large Language Models (LLMs) have revolutionized protein engineering by capturing complex sequence patterns from extensive databases such as UniProt, enabling their deployment for a plethora of applications such as protein secondary structure prediction and functional annotation (Ferruz and Birte, 2022; Valentini et al., 2023; The UniProt Consortium T, 2018). In this context, *Finenzyme* was introduced as a framework that leverages conditional transfer learning (Madani et al., 2023) to generate enzyme sequences corresponding to specific Enzyme Commission (EC) numbers, demonstrating that fine-tuning pre-trained models on specific enzymatic datasets improves prediction and generation of enzymes compared to general purpose Protein Language Model ProGen (Nicolini et al., 2025). In this work, we introduce an *in-silico* selection process to identify the *Finenzyme* generated sequences that best preserve or possibly enhance the functional characteristics of specific EC categories. The selected sequences are then subjected to molecular dynamics (MD) simulations, providing a dynamic evaluation of their structural stability and potential catalytic behavior. In particular, the stability, folding, and potential catalytic activity of the analyzed structures were evaluated monitoring the root mean square deviation (RMSD), root mean square fluctuation (RMSF), radius of gyration (RoG), and solvent-accessible surface area (SASA) of alpha carbons (Cα) throughout MD simulations (K and Venugopal, 2024). RMSD evaluates system convergence and stability, RMSF measures residue fluctuations, RoG indicates protein compactness (with higher values suggesting decreased stability), and SASA reflects solvent exposure, where excessively high values can impair catalytic competence. By combining conditional pre-trained language model (PLM) fine-tuning and enzyme selection with detailed dynamic analysis via MD simulations, our approach extends the application of sequence-based predictions to encompass the conformational landscapes that underlie enzyme activity, an important aspect for both biomedical and industrial applications (Walsh and Walsh, 2022). We emphasise that the objective is sequence-space exploration within established enzyme families rather than the design of novel folds or new catalytic functions: the pipeline is intended to identify sequences that diverge substantially from natural enzymes in sequence space while retaining a stable, native-like fold, possibly improving the stability and enzymatic activity of their natural counterparts. Figure 1 summarizes the overall framework of this project.

## 2 Materials and methods

Finenzyme’s PLM (Section 2.1) has been utilized to generate new artificial sequences that catalyze specific enzymatic reactions (Section 2.2). An additional enrichment step has been employed to select only the most novel, high-quality and 3D structure preserving candidates (Section 2.3). The selected sequences were then characterized and further validated through MD simulations (Section 2.4).

### 2.1 Model for sequence generation

Finenzyme is a fine-tuning procedure which builds on the ProGen Conditional Transformer Language Model (CTRL) (Madani et al., 2023; Nicolini et al., 2025). ProGen adapts the CTRL architecture for autoregressive protein sequence generation and uses pre-pended tags that denote functional or taxonomic attributes to guide the generative process (Keskar et al., 2019). Finenzyme’s fine-tuning preserves the knowledge learned by the pre-trained model over a large, diverse protein datasets, while shifting its generating capabilities towards more specific protein groups. In particular, Finenzyme has been fine-tuned on enzyme classes that catalyze the same reaction and further details on the architecture, training strategies, and evaluation protocols can be found in our previous work (Nicolini et al., 2025). This strategy is computationally inexpensive yet highly effective as it significantly boosts the pre-trained model’s performance in both sequence prediction and sequence generation.

### 2.2 Datasets

In our previous article, we showed that Finenzyme’s fine-tuning works best on enzyme groups with well-defined catalytic reactions (Nicolini et al., 2025). Therefore, we generated and selected enzymes belonging to four specific EC categories (1.1.1.1, 2.1.1.37, 5.4.99.5, and 6.3.4.15, corresponding to alcohol dehydrogenase, DNA(cytosine-5)-methyltransferase, chorismate mutase, and biotin-protein ligase enzymes, respectively) to provide a dynamic evaluation of their structural stability. The enzymes belonging to the EC categories of interest were curated from UniProt (The UniProt Consortium T, 2018). Furthermore, redundant sequences were removed as well as sequences shorter than 10 (considered as partial reads) or longer than 500 amino acids (because of Finenzyme’s maximum input size). For each specific EC category, we performed generation using nucleus sampling [details in Nicolini et al., 2025] with sampling parameters *p* = 0.5 and *p* = 0.75; higher values of *p* allow the model greater freedom in sequence generation.

### 2.3 Selection of generated sequences

Although Finenzyme’s sequence generation is computationally inexpensive, the stochastic sampling can yield degenerate or repetitive sequences. To select non redundant, high-quality and tertiary structure preserving sequences, we applied the following three filters:

#### 2.3.1 Novelty/non-redundancy filter

Using BLASTp, we computed for each generated sequence its maximum identity (MaxID) to the curated UniProt set for the corresponding EC number (the same data used for fine-tuning) and excluded any sequence sharing >80% identity. This both removes near-duplicates and quantifies how far each retained sequence lies from the sequences seen during training.

#### 2.3.2 Structure-quality filter

We predicted each sequence’s tertiary structure using ESMFold and retained only those achieving a prediction-confidence score (pLDDT) > 0.8 (0.7 is already considered good by ESMFold’s authors) (Lin et al., 2023).

#### 2.3.3 Fold confirmation filter

We used Foldseek (van Kempen et al., 2024) to query the PDB with each predicted structure, discarding any sequence whose most similar natural structure had a TM-score <0.8, target coverage <0.9, and query coverage <0.9. This ensures the final candidates have a plausible overall fold.

### 2.4 Molecular dynamics simulations

For each EC category, a randomly selected subset of the generated sequences was used as input for the Basic Local Alignment Search Tool (BLAST), which compares a query sequence against a reference database to identify the closest homologs (Altschul et al., 1990). The goal was to determine the experimentally resolved 3D protein structure most similar to Finenzyme’s generated sequences. To this end, we queried the “*Protein Data Bank (PDB) proteins*” database using the blastp algorithm. The resulting PDB structure was then structurally aligned against all generated sequences within the same EC class to confirm that it provided a robust and coherent 3D scaffold for downstream MD simulations. This structure was ultimately selected as the reference model for evaluating whether the generated sequences adopted conformations comparable to those observed in experimentally determined X-ray structures. When PDB structures contained additional ligands besides the protein, only the cofactor was retained for MD simulations. The cofactor was incorporated into all selected generated models by structural alignment to the corresponding reference crystals. Owing to the high conservation of the active-site regions, cofactors could be directly transferred to the aligned positions without steric conflicts. Each resulting protein/cofactor complex was then locally optimized and energy-minimized using Maestro (release 2024–4, Schrödinger, LLC, New York, NY, USA) to relieve residual strain and optimize side-chain conformations prior to MD simulations. These were performed on a randomly selected subset of generated sequences to avoid over-representation of individual targets and reduce bias across enzyme classes, maintaining similar absolute numbers of trajectories per target rather than uniform sampling fractions. In fact, in the case of EC 6.3.4.15 (*n* = 324), MD simulations were capped to match other classes due to the otherwise disproportionate workload. In particular, the selected protein structures were subjected to a single 250 ns MD simulation using Amber24 software. As only one trajectory was generated for each system, the results may be influenced by the stochastic nature of MD simulations. Nevertheless, all systems were treated consistently under identical simulation conditions, allowing for meaningful comparative analysis between them. Simulations were run with the GPU-accelerated PMEMD code using a 2 fs time step and were performed in a TIP3P-solvated system with counter-ions for neutrality, employing the *ff14SB* force field for protein atoms (Maier et al., 2015). To remove initial atomic clashes, the systems underwent a multi-step relaxation protocol involving energy minimization, gradual heating to 300 K, and equilibration at 1 atm. More details of the MD simulations are provided in the Supplementary materials. MD trajectory analyses of Cα RMSD, RMSF, RoG, and SASA were performed using AmberTools24, considering only the protein residues aligned with the reference protein to enable more accurate comparative values.

### 2.5 Statistical analysis

To reproducibly classify Finenzyme-generated sequences as stable or potentially catalytically competent, threshold-based criteria were applied. Since the analyzed enzymes differ in size, fold, and reference PDB structure, we did not apply a single absolute cutoff across all systems. Instead, we used reference-normalized thresholds derived from the corresponding MD simulation of the experimentally determined reference protein. For each generated sequence, RMSD, RMSF, RoG, and SASA were computed over the same aligned Cα residues used for the reference system. Although the *μ* + 2*σ* criterion (where *μ* is the mean and *σ* the standard deviation) is commonly adopted in statistical practice, encompassing ~95% of observations under the assumption of a normal distribution and thus serving as a standard approach for identifying non-anomalous values, our goal extended beyond simple outlier exclusion. Instead, this study aims to select structures that are highly representative of the reference conformational ensemble, thereby enabling a more stringent assessment of Finenzyme’s ability to generate structurally meaningful enzyme variants. For this reason, a stricter threshold of *μ* + *σ* was employed for RMSD and RMSF values. This approach, capturing ~68% of the data, preferentially retains conformations closely aligned with the central tendency of the distribution, thereby enforcing higher structural similarity while reducing the inclusion of marginally deviating configurations. In contrast, for RoG and SASA values, the *μ* + 2*σ* criterion was applied. This choice was motivated by the very low standard deviation values observed for these metrics, which would otherwise lead to the exclusion of sequences exhibiting values close to the reference and objectively favorable from a structural standpoint.

A generated protein was classified as stable/folded if:

- mean C*α*-RMSD ≤ *μ*_ref + *σ*_ref;

while it was classified as potential catalytically competent if it satisfied all of the following criteria over the equilibrated portion of the trajectory:

- mean C*α*-RMSF ≤ *μ*_ref + *σ*_ref;
- mean C*α*-RoG ≤ *μ*_ref + 2*σ*_ref;
- mean C*α*-SASA ≤ *μ*_ref + 2*σ*_ref.

Only in the case of 1ODE (EC 5.4.99.5), the *μ* + 2*σ* criterion was extended to RMSD and RMSF, due to the exceptionally low reference mean values (*μ*_ref = 1.06 Å and 0.73 Å, respectively), which would have rendered stricter thresholds overly restrictive.

The complete list of the numeric cutoffs applied for each reference is reported in the Supplementary Table S1.

## 3 Results and discussion

In Table 1 the initial non-duplicated Finenzyme-generated sequences (for each specific EC number considered) are presented, together with the resulting sequences after the selection process described in the Materials and Method section 2.3.

**Table 1:** Generated and filtered enzymes for different EC categories.

EC	*p* = 0.5	*p* = 0.75
1.1.1.1	1/1	715/189
2.1.1.37	698/13	925/15
5.4.99.5	318/54	923/91
6.3.4.15	390/150	867/174

The number pairs *x*/*y* in the second and third column represent, respectively, the number of unique generated sequences from a set of 1,000 for each EC and the number of sequences after the filtering process. *p* denotes the nucleus sampling value.

**Figure 2:**
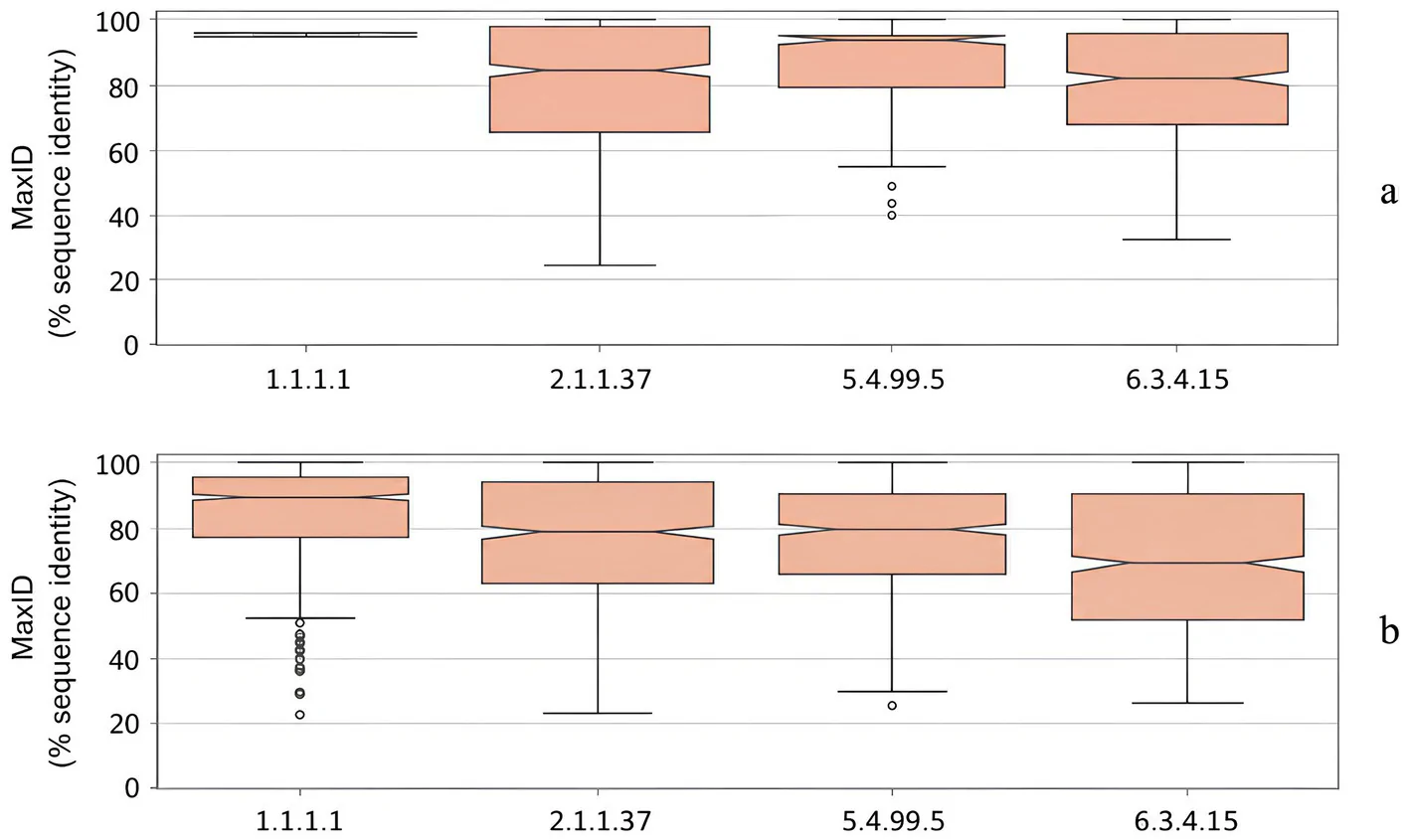
Distribution of the maximum BLASTp sequence identity (MaxID) of each Finenzyme-generated sequence to the UniProt set of the corresponding EC class. **(a)** nucleus sampling *p* = 0.5; **(b)** *p* = 0.75.

Figure 2 reports the distribution of maximum BLASTp identity (MaxID) between each generated sequence and the EC-specific UniProt set, for nucleus sampling *p* = 0.5 (Figure 2a) and *p* = 0.75 (Figure 2b). By filtering, all retained sequences fall below the 80% threshold, but the distributions show that most are well below it, confirming that the model produces sequences meaningfully divergent from the training data rather than reproducing existing entries; greater sampling freedom (*p* = 0.75) shifts the distribution toward lower identities. Then, we performed MD simulations to determine whether sequences generated by Finenzyme could produce folded and stable enzymes with the potential to maintain the catalytic activity. Because MD simulations were run only on candidates passing the novelty, structure-quality, and fold-confirmation filters, the success rates reported below describe this pre-selected subset and are not intended as a measure of the raw generative output of Finenzyme. RMSD, RMSF, RoG, and SASA were calculated over the entire simulation time, and structures generated with sampling parameter *p* = 0.5 and *p* = 0.75 were analyzed separately to assess the impact of different nucleus filtering thresholds.

### 3.1 EC 1.1.1.1 (alcohol dehydrogenase)

**Figure 3:**
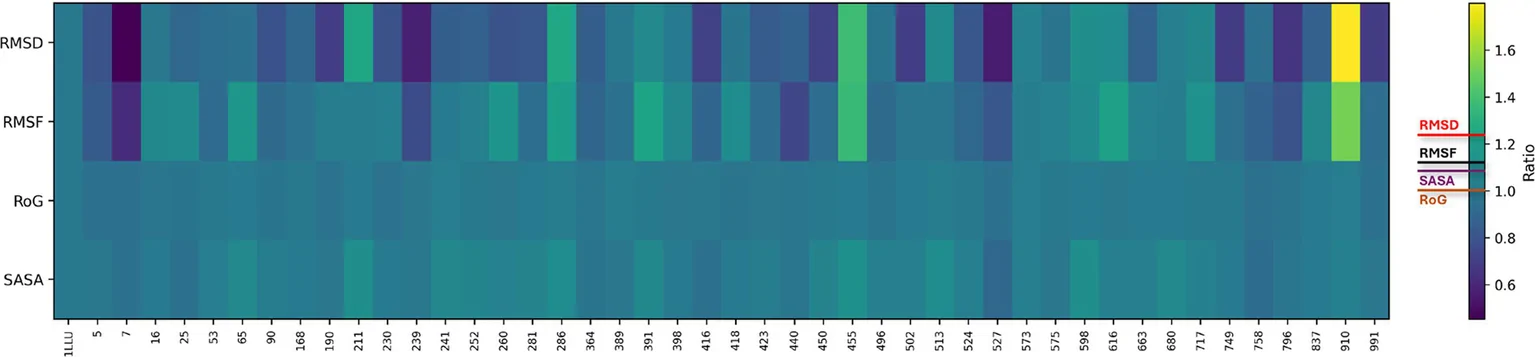
Heatmap plot showing the ratios of RMSD, RMSF, RoG, and SASA for each sequence relative to the reference structure 1LLU. Values are normalized by dividing each parameter by its corresponding value in 1LLU, with the colour scale representing the magnitude of the resulting ratio. In the ratio panel, the threshold ratios of the MD metrics are shown in different colors.

For EC 1.1.1.1, Finenzyme generated only one structure with *p* = 0.5 and 189 with *p* = 0.75 (Table 1), consequently only the latter were considered. Of these, 45 out of 189 structures were randomly selected (corresponding to 23.8% of the total) for MD simulations. The structure with PDB accession code 1LLU (chain A) was used as reference, which features the ternary complex of alcohol dehydrogenase enzyme of *Pseudomonas aeruginosa* with coenzyme nicotinamide adenine dinucleotide (NAD^+^) and ethylene glycol (Levin et al., 2004). As reported in the Supplementary Table S2 and illustrated in Figure 3, four structures (211, 286, 455 and 910) exhibited an average RMSD higher than threshold (3.55 Å), indicating lower stability during MD simulations compared to the reference. Variant 910, in particular, displayed clear unfolding. Three of these structures, with two additional variants (391 and 616), exhibit globally higher RMSF values than threshold (1.39 Å). This may indirectly affect catalytic competence by altering global conformational dynamics or transmitting structural perturbations to the active site, suggesting a higher risk of reduced potential enzymatic competence. Interestingly, all RoG values fall within the threshold (21.6 Å), indicating that the global compaction of every variant is preserved and comparable to the reference structure. Regarding SASA, five structures exhibit values exceeding the threshold (17,110 Å^2^), which may interfere with their potential catalytic competence. Three of these (211, 286, and 455) coincide with variants showing low stability and partial unfolding. The remaining two structures (513 and 598) display RMSD, RMSF, and RoG values within threshold but elevated SASA, suggesting locally increased surface exposure despite preserved global stability. This pattern may indicate excessive surface exposure that can distort active-site geometry, promote non-specific solvent interactions, or reflect partial destabilization of the enzyme, all of which increase the risk of reduced or loss of potential enzymatic activity.

Overall, 41/45 models (91.1%) maintained proper folding and stability, while 37/45 (82.2%) exhibit structural compatibility with catalysis competence (Table 2).

**Table 2:** Percentage of stable/folded and potentially catalytically competent EC 1.1.1.1 enzymes generated (resembling 1LLU) with *p* = 0.75 based on the results of the performed MD simulations.

EC 1.1.1.1 (1LLU)	*p* = 0.75 (total)
Stable/folded	41/45 (91.1%)
Potentially catalytically competent	37/45 (82.2%)

**Figure 4:**
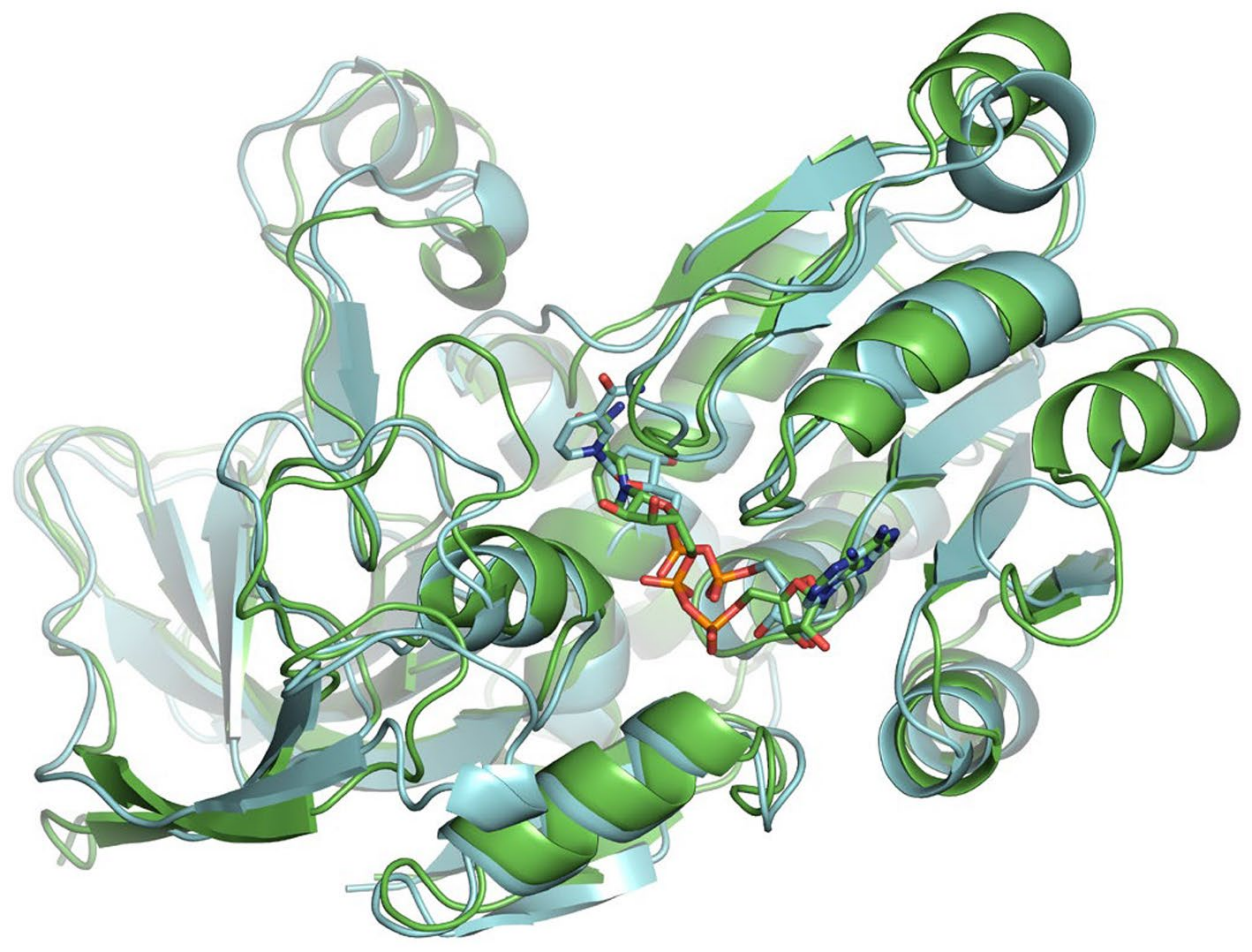
Representative alignment of Finenzyme-generated EC 1.1.1.1 structure 7 (cyan) aligned with the crystal structure of the *Pseudomonas aeruginosa* alcohol dehydrogenase enzyme (PDB accession code: 1LLU, green) after 250 ns MD simulation. The excellent structural overlap with the reference indicates that Finenzyme generated highly stable enzymes.

As an illustrative example, we present here the structure of one of the most promising Finenzyme-generated structures of EC 1.1.1.1, stabilized over the simulations (structure 7, Supplementary Table S2), aligned with the crystal structure of 1LLU after 250 ns MD simulation (Figure 4). It is observable how the tertiary structure of the generated sequences with Finenzyme (in this case structure 7) aligns closely with the reference protein (RMSD = 1.7 Å), thereby preserving excellent folding and stability. Consistent with the MD simulation trajectories, the models show structural features suggestive of retained catalysis.

### 3.2 EC 2.1.1.37 (DNA(cytosine-5)-methyltransferase)

**Figure 5:**
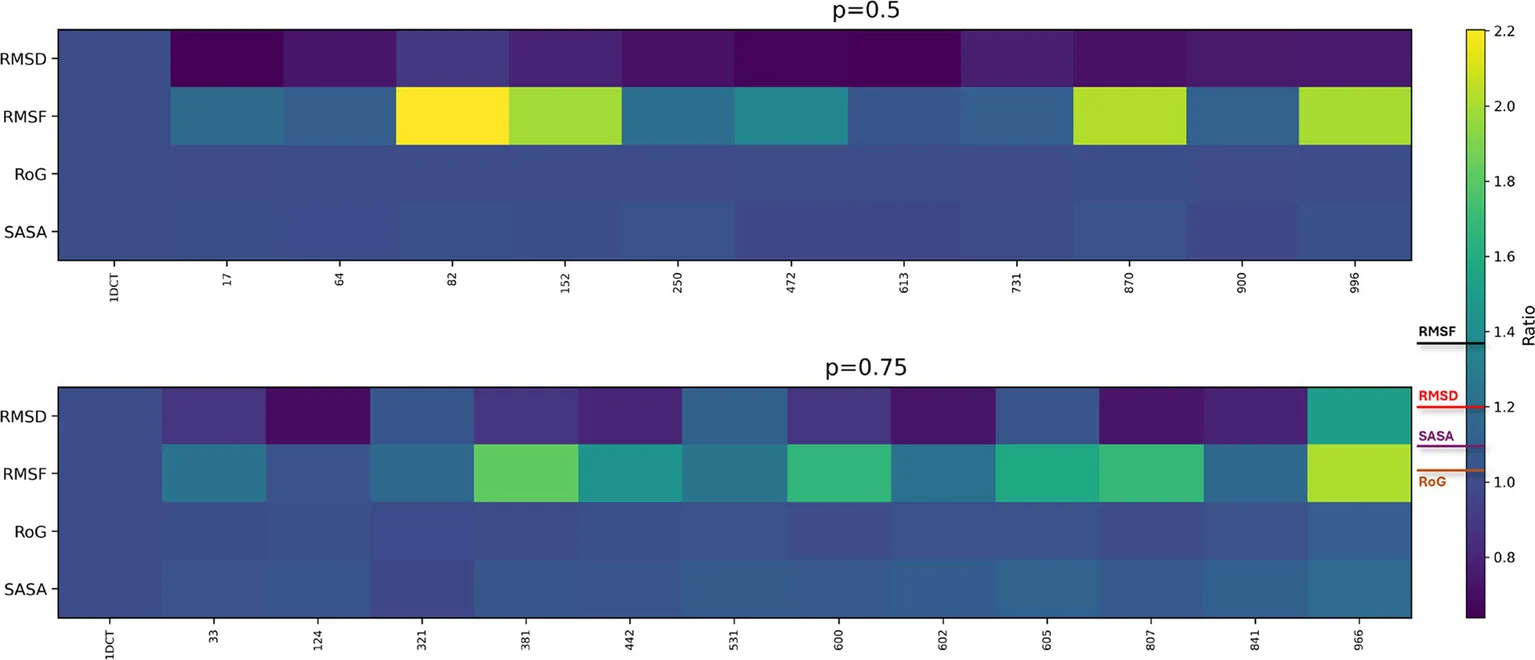
Heatmap plot showing the ratios of RMSD, RMSF, RoG, and SASA for each structure generated with *p* = 0.5 (top) and *p* = 0.75 (bottom) relative to the reference 1DCT. Values are normalized by dividing each parameter by its corresponding value in 1DCT, with the colour scale representing the magnitude of the resulting ratio. In the ratio panel, the threshold ratios of the MD metrics are shown in different colors.

In the case of EC 2.1.1.37, the structure with PDB accession code 1DCT was identified as the most similar, which features the enzyme of *Haemophilus influenzae aegyptius* covalently bound with DNA, and used as reference (Reinisch et al., 1995). However, it is important to note that 1DCT consists of 324 residues, whereas the generated sequences comprise a range of residues mainly around 340. Nevertheless, the structures were perfectly superimposable onto the crystallographic one, except for few residues at the C-terminus. In this case, to ensure a reliable comparison with a crystallographic reference structure, only the 324 residues that aligned perfectly with 1DCT were considered when calculating the structural stability parameters. Sequences 367, 726 (*p* = 0.5) and 399, 678, 890 (*p* = 0.75) were exceptionally long (~400 residues), poorly superimposable on 1DCT, and most similar by BLAST to a *Winogradskyella sediminis* enzyme lacking the crystal structure. Consequently, they were excluded from MD analysis. As shown in Figure 5, Supplementary Table S3, all generated EC 2.1.1.37 enzyme models exhibited stable convergence, with mean RMSD values even lower than those of the reference structure 1DCT, indicating robust stability throughout the MD simulations. The only exception was structure 966 (*p* = 0.75), which underwent unfolding. RMSF analysis revealed four structures generated with *p* = 0.5 that exceeded the threshold of 1.09 Å (82, 152, 870, and 996), whereas six variants generated with *p* = 0.75 surpassed this threshold (381, 442, 600, 605, 807, and 966). Although their RMSF profiles are closely aligned with that of the reference, the markedly elevated peaks may alter the global conformational dynamics of the enzyme, potentially leading to reduced, or even loss, of enzymatic competence. Similarly to what was observed for the protein belonging to EC 1.1.1.1, none of the analyzed structures exhibited RoG values exceeding the threshold (21.0 Å). The only exception is structure 966 (*p* = 0.75), which further supports the observation that it underwent progressive unfolding during MD simulation. Notably, none of the structures generated with *p* = 0.5 exceeded the thresholds for RoG or SASA (21.0 Å and 14,946 Å^2^, respectively). In contrast, within the *p* = 0.75 set, structure 966 exceeded both thresholds, while variants 605 and 841 exhibited elevated SASA values only.

Consequently, at *p* = 0.5, all 11 models maintained proper folding and stability (100%), while at *p* = 0.75, one model (966) failed (11/12 = 91.7% success rate). Regarding the potential catalytic competence of the generated enzymes, anomalous regional dynamics (as indicated by RMSF) and/or significantly elevated SASA values suggest a high probability of functional impairment. Consequently, only 7/11 (63.6%) of *p* = 0.5 models and 5/12 (41.7%) of *p* = 0.75 models are likely to retain structural characteristics compatible with enzymatic activity. The results are resumed in Table 3.

**Table 3:** Percentage of stable/folded and potentially catalytically competent EC 2.1.1.37 enzymes generated (resembling 1DCT) with *p* = 0.5 and *p* = 0.75 based on the results of the performed MD simulations.

EC 2.1.1.37 (1DCT)	*p* = 0.5	*p* = 0.75	Total
Stable/folded	11/11 (100%)	11/12 (91.7%)	22/23 (95.6%)
Potentially catalytically competent	7/11 (63.6%)	5/12 (41.7%)	12/23 (52.2%)

In this case, it is observable that structures generated by Finenzyme using a sequence-generation model with reduced freedom (*p* = 0.5) were markedly more stable and exhibited overall improved conformational dynamics compared with those produced under higher freedom conditions (*p* = 0.75) (Table 3).

### 3.3 EC 5.4.99.5 (chorismate mutase)

For the generated enzymes belonging to the EC 5.4.99.5 class, two distinct types of proteins were identified based on structural similarity: those resembling the protein with accession code 3RMI (*Bartonella henselae strain Houston-1*) and those resembling 1ODE (*Thermus thermophilus HB8*). The PDB entry 3RMI contains chorismate mutase complexed with D-malate, along with glycerol and 1,2-ethanediol in solution, whereas PDB entry 1ODE contains only the protein. Of the 54 structures generated with *p* = 0.5, 48 were found to be superimposable with 3RMI, and, given the large number of data set, 16 randomly selected models (representing 33% of the total) were subjected to MD simulations and analysis (Supplementary Tables S4-, S5-top). For *p* = 0.75, among the 91 generated structures, only 21 were superimposable with 3RMI and, in this case, all the structures were considered for the analysis through MD simulations (Supplementary Tables S4-, S5-bottom).

**Figure 6:**
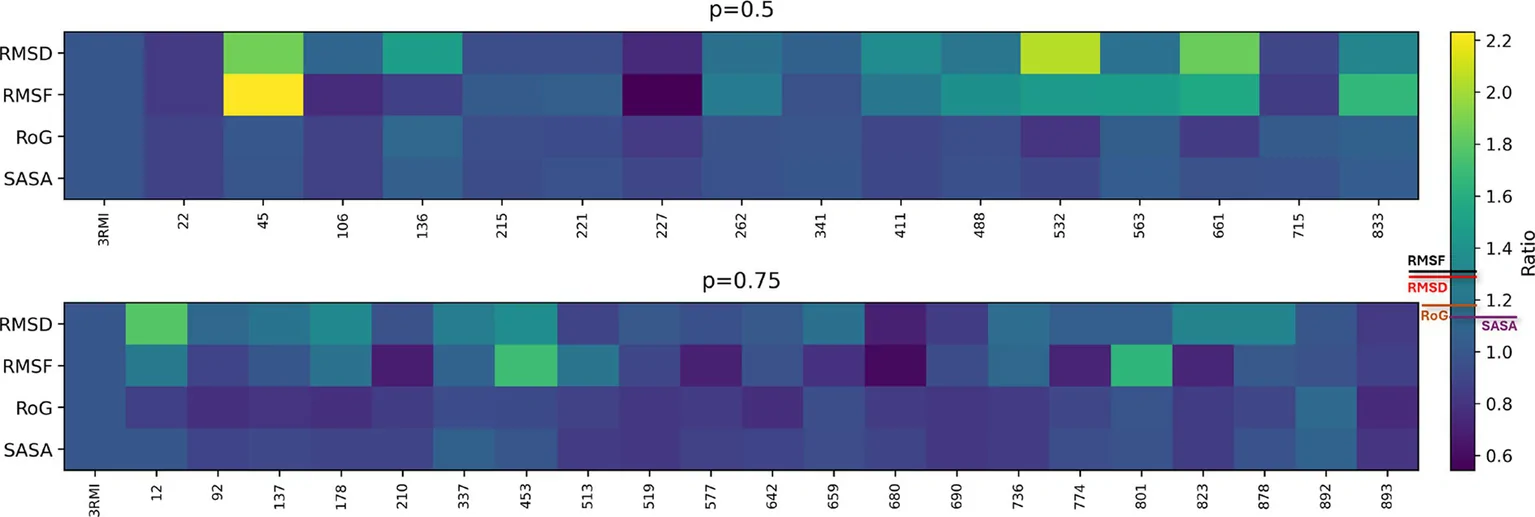
Heatmap plot showing the ratios of RMSD, RMSF, RoG, and SASA for each structure generated with *p* = 0.5 (top) and *p* = 0.75 (bottom) relative to the reference structure 3RMI. Values are normalized by dividing each parameter by its corresponding value in 3RMI, with the colour scale representing the magnitude of the resulting ratio. In the ratio panel, the threshold ratios of the MD metrics are shown in different colors.

Regarding the chorismate mutase enzyme of *Bartonella henselae strain Houston-1*, 10 out of 16 structures generated with *p* = 0.5 (68.8%) exhibited excellent stability, as indicated by RMSD values comparable to, or even slightly lower than, those of 3RMI across all evaluated parameters, while this was observed for 18/21 structures (85.7%) generated with *p* = 0.75 (Figure 6). Concerning the potential catalytic competence, the number of suitable structures decreases to 8/16 (50.0%) and 17/21 (80.9%) for *p* = 0.5 and *p* = 0.75, respectively, as structures 488, 563 and 801 exhibited excessive residue fluctuations than threshold (3.87 Å) that were not consistent with the reference structure 3RMI (Figure 6). Interestingly, most structures exhibited lower RoG values compared to 3RMI, suggesting a greater tendency to adopt more compact and globular conformations. This structural feature could have important industrial implications, as increased compactness is often associated with enhanced structural stability, which may translate into improved tolerance to harsh industrial conditions (Figure 6). The results are resumed in Table 4.

**Table 4:** Percentage of stable/folded and potentially catalytically competent EC 5.4.99.5 enzymes generated (resembling 3RMI) with *p* = 0.5 and *p* = 0.75 based on the results of the performed MD simulations.

EC 5.4.99.5 (3RMI)	*p* = 0.5	*p* = 0.75	Total
Stable/folded	10/16 (62.5%)	18/21 (85.7%)	28/37 (75.7%)
Potentially catalytically competent	8/16 (50.0%)	17/21 (80.9%)	25/37 (67.6%)

Contrary to the trend observed in the previous EC classes, sequences generated with greater sequence freedom (*p* = 0.75) displayed superior folding stability and stronger signatures of potential catalytic competence than those produced with reduced freedom (*p* = 0.5), as suggested by RMSF analysis. For folding stability, 18/21 sequences (85.7%) at *p* = 0.75 were stable versus 10/16 (62.5%) at *p* = 0.5. Similarly, RMSF-based indicators of potential catalytic competence were observed in 17/21 sequences (80.9%) at *p* = 0.75 compared with 8/16 (50.0%) at *p* = 0.5 (Table 4).

**Figure 7:**
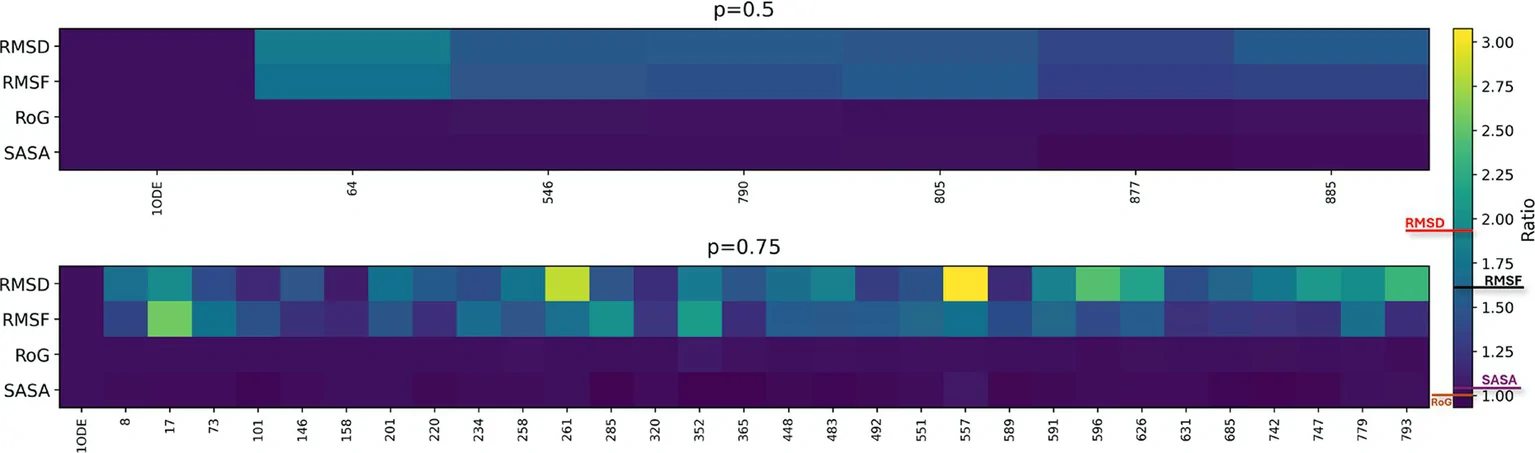
Heatmap plot showing the ratios of RMSD, RMSF, RoG, and SASA for each structure generated with *p* = 0.5 (top) and *p* = 0.75 (bottom) relative to the reference structure 1ODE. Values are normalized by dividing each parameter by its corresponding value in 1ODE, with the colour scale representing the magnitude of the resulting ratio. In the ratio panel, the threshold ratios of the MD metrics are shown in different colors.

Regarding the chorismate mutase enzyme of *Thermus thermophilus HB8*, of the 54 structures generated with *p* = 0.5, only 6 were found to be superimposable with 1ODE (Supplementary Table S5-top) while of the 70 structures generated with *p* = 0.75, approximately 40% (30 models) were randomly selected as a representative sample and subjected to MD simulations (Supplementary Table S5-bottom). Notably, as represented in Figure 7-top, all the structures generated with *p* = 0.5 (6/6) showed MD metrics below their respective thresholds (see Materials and Methods for details). In the case of those generated with *p* = 0.75, structures 17, 261, 557, 596, 626, 747, 779 and 793, exhibited average RMSD values higher than threshold of 2.02 Å (Figure 7-bottom), reducing the percentage of stable sequences to 73.3% (22/30) (Table 5). Beyond the eight unstable (and thus potentially non-competent) structures, five sequences (234, 285, 352, 551, and 591) show RMSF values exceeding the 1.17 Å threshold, further reducing the pool of potentially catalytically competent structures to 17/30 (56.7%). RoG and SASA values generally remain within their respective thresholds, except for variant 352, which exhibits reduced compactness and a RoG value above the cutoff.

**Table 5:** Percentage of stable/folded and potentially catalytically competent EC 5.4.99.5 enzymes generated (resembling 1ODE) with *p* = 0.5 and *p* = 0.75 based on the results of the performed MD simulations.

EC 5.4.99.5 (1ODE)	*p* = 0.5	*p* = 0.75	Total
Stable/folded	6/6 (100%)	(22/30 = 73.3%)	28/36 (77.8%)
Potentially catalytically competent	6/6 (100%)	(17/30 = 56.7%)	23/36 (63.9%)

Overall, considering all the EC 5.4.99.5 enzymes generated, the majority of models showed smooth convergence and close agreement with their respective references. In fact, 58 out of 73 structures (79.4%) exhibited stable folding throughout the MD simulations, while 48 out of 73 (65.7%) are predicted to adopt conformations potentially compatible with catalytic activity, based on the limited analysis of MD-derived average RMSF, RoG, and SASA values.

### 3.4 EC 6.3.4.15 (biotin-protein ligase)

Two distinct types of generated enzymes within the EC 6.3.4.15 were identified based on structural similarity: those resembling the protein with PDB accession code 1WNL (*Pyrococcus horikoshii OT3*) and those resembling 6NDL (*Staphylococcus aureus*) (Bagautdinov et al., 2005; Lee et al., 2019). PDB entry 1WNL contains the enzyme complexed with the cofactor adenosine-5′-diphosphate (ADP), whereas PDB entry 6NDL comprises biotin protein ligase bound to a sulfonamide inhibitor. Of the 150 structures generated at *p* = 0.5, we randomly selected 49 (32.7%) as a representative sample. Among these, 28 superimposed well onto 1WNL and 21 onto 6NDL (Supplementary Tables S6-, S7-top). In the case of *p* = 0.75, we randomly selected the 26.4% of the generated structures (46/174) as a representative sample. Among these, 30 resemble the structure of 1WNL and 16 of 6NDL (Supplementary Tables S6-, S7-bottom).

**Figure 8:**
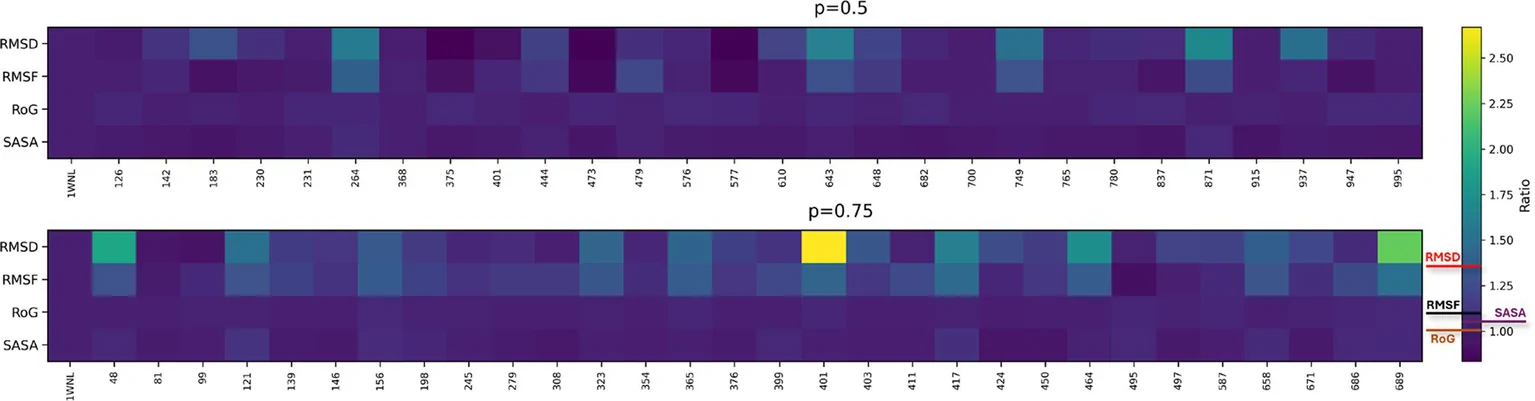
Heatmap plot showing the ratios of RMSD, RMSF, RoG, and SASA for each structure generated with *p* = 0.5 (top) and *p* = 0.75 (bottom) relative to the reference structure 1WNL. Values are normalized by dividing each parameter by its corresponding value in 1WNL, with the colour scale representing the magnitude of the resulting ratio. In the ratio panel, the threshold ratios of the MD metrics are shown in different colors.

For the biotin-protein ligase from *Pyrococcus horikoshii OT3*, 23 out of 28 structures generated with *p* = 0.5 (82.1%) exhibited excellent stability, with values comparable to or slightly lower than those of 1WNL across all evaluated parameters (Figure 8-top), whereas this percentage decreased to 66.7% (20/30 structures) for *p* = 0.75 (Figure 8-bottom). Notably, comparable percentages are observed for potential catalytic competence, as the same variants identified as poorly stable or partially unfolded in MD simulations also exhibit RMSF values above the threshold (1.10 Å), with the only exception being sequence 479 (*p* = 0.5), which is additionally included. Conversely, RoG and SASA values remain within their respective thresholds (17.9 Å and 12,154 Å^2^, respectively) for all variants, except for 121 and 417 (*p* = 0.75), which are likewise classified as unstable (Figure 8). The results are resumed in Table 6.

**Table 6:** Percentage of stable/folded and potentially catalytically competent EC 6.3.4.15 enzymes generated (resembling 1WNL) with *p* = 0.5 and *p* = 0.75 based on the results of the performed MD simulations.

EC 6.3.4.15 (1WNL)	*p* = 0.5	*p* = 0.75	Total
Stable/folded	23/28 = 82.1%	20/30 = 66.7%	43/58 (74.1%)
Potentially catalytically competent	22/28 = 78.6%	20/30 = 66.7%	42/58 (72.4%)

**Figure 9:**
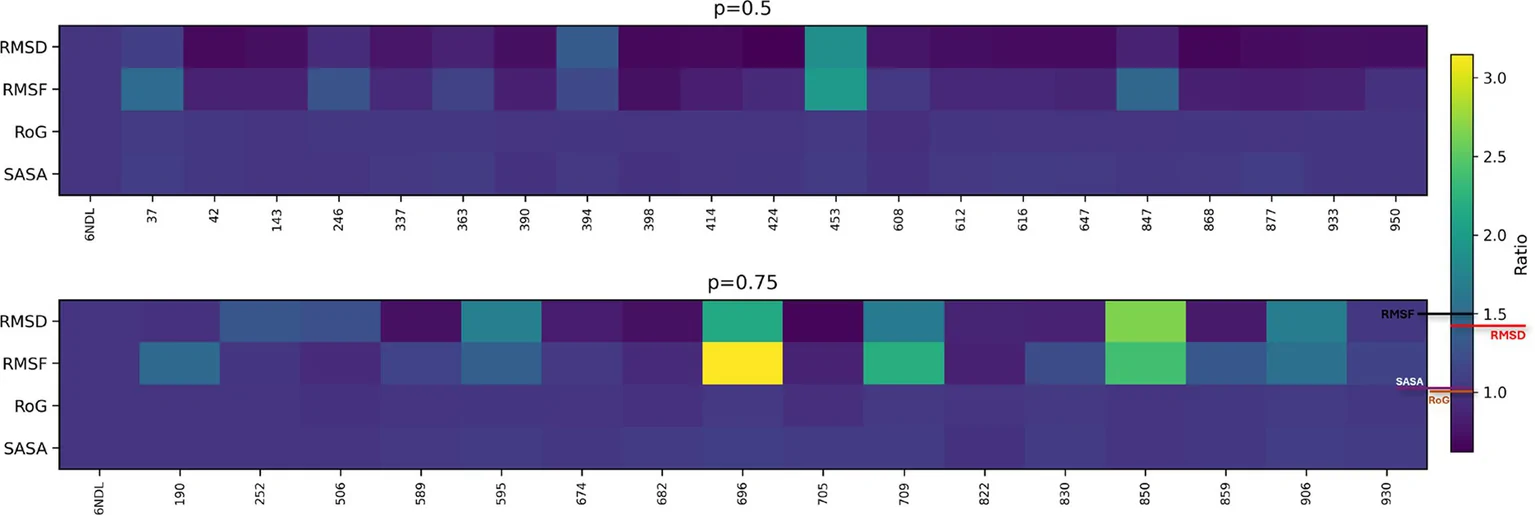
Heatmap plot showing the ratios of RMSD, RMSF, RoG and SASA for each structure generated with *p* = 0.5 (top) and *p* = 0.75 (bottom) relative to the reference structure 6NDL. Values are normalized by dividing each parameter by its corresponding value in 6NDL, with the colour scale representing the magnitude of the resulting ratio.

Analysis of the biotin-protein ligase from *Staphylococcus aureus* shows that only one of the generated structures (453) with *p* = 0.5 (20/21 = 95.2%) displayed higher average RMSD value than threshold (4.90 Å), while the others showed values similar to or even below that of reference 6NDL (Figure 9-top; Supplementary Table S7-top). Also in this case, this success rate dropped to 68.7% (11/16) for the sequences generated with *p* = 0.75 (Figure 9-bottom; Supplementary Table S7-bottom). For *p* = 0.5, four additional variants (37, 363, 616, and 877) exceed the SASA threshold (16,312 Å^2^), suggesting an increased risk of structural destabilization for the potential catalytic competence and decreasing the proportion to 76.2% (16/21). For *p* = 0.75, three additional sequences (682, 830, and 930) similarly exceed the threshold, further lowering the percentage in that case to 50.0% (8/16). The results are resumed in Table 7.

**Table 7:** Percentage of stable/folded and potentially catalytically competent EC 6.3.4.15 enzymes generated (resembling 6NDL) with *p* = 0.5 and *p* = 0.75 based on the results of the performed MD simulations.

EC 6.3.4.15 (6NDL)	*p* = 0.5	*p* = 0.75	Total
Stable/folded	20/21 = 95.2%	11/16 = 68.7%	31/37 (83.8%)
Potentially catalytically competent	16/21 = 76.2%	8/16 = 50.0%	24/37 (64.9%)

As shown in Table 7, structures generated by Finenzyme with a lower degree of sequence variability (*p* = 0.5) display substantially higher stability and improved conformational behavior compared with those generated under higher variability conditions (*p* = 0.75). Specifically, the proportion of stable/folded structures decreases from 95.2% (20/21) at *p* = 0.5 to 68.7% (11/16) at *p* = 0.75, accompanied by a reduction in potentially catalytically competent variants from 76.2% (16/21) to 50.0% (8/16).

In summary, all the generated EC 6.3.4.15 enzymes predominantly achieved smooth convergence and strong alignment with their respective reference structures. Specifically, 74/95 models (77.9%) demonstrated stable folding over the course of MD simulations, and 66/95 (69.5%) are forecasted to preserve the potential catalytic competence based on the limited computational analysis performed, considering only average RMSF, RoG, and SASA values.

### 3.5 Distribution of data across EC classes

**Figure 10:**
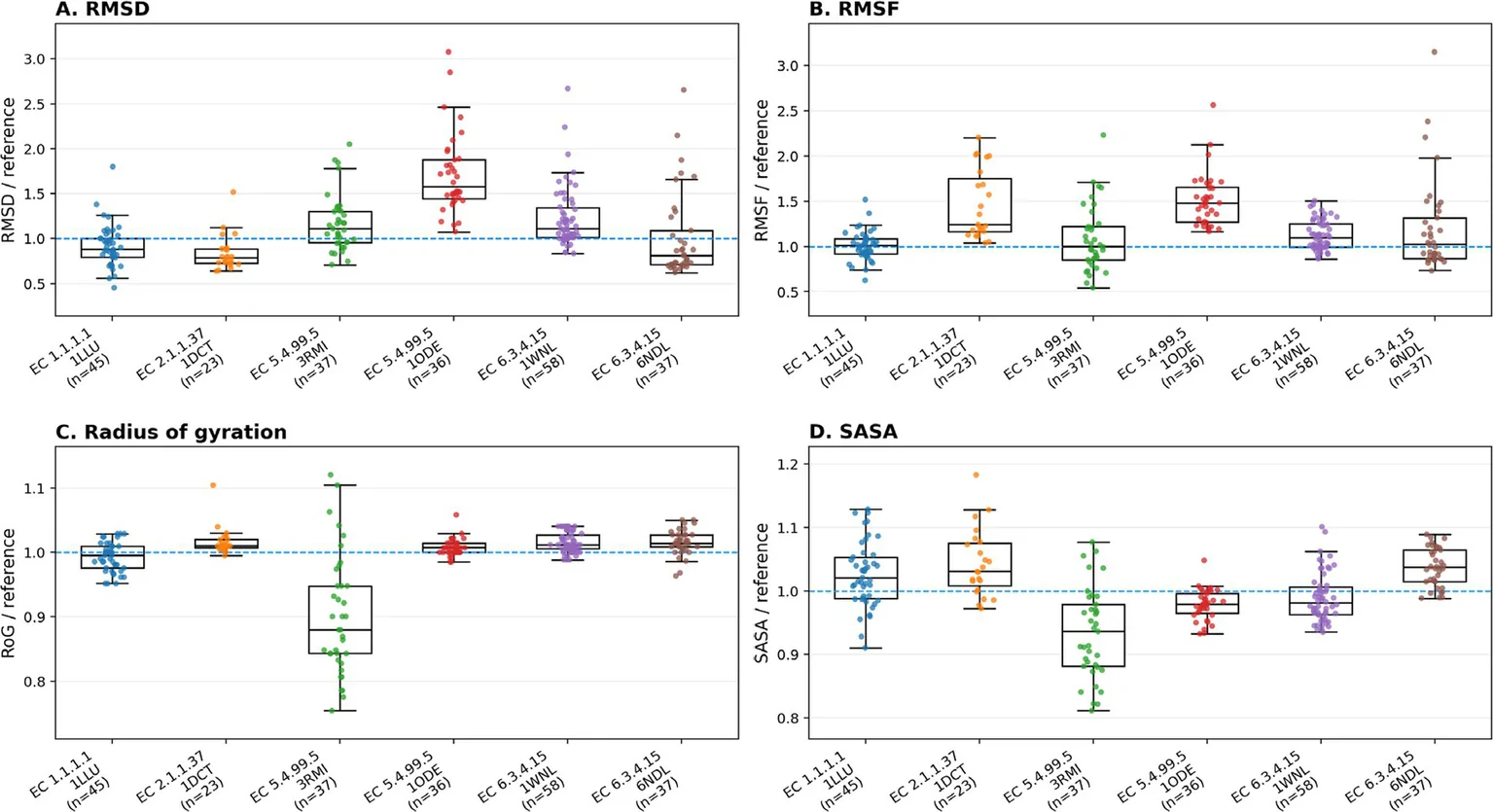
MD descriptor distributions of **(A)** *RMSD*, **(B)** *RMSF*, **(C)** *RoG*, and **(D)** *SASA* ratios for generated enzyme conformers, stratified by EC class and benchmarked against their respective crystallographic reference structures. Ratios were computed by normalizing each MD-derived descriptor to the corresponding experimental value. Cyan dashed line denotes the reference value (*ratio* = 1). Points represent individual generated models, while boxplots capture the intra-class dispersion and descriptor variability.

To conclude our analysis, we evaluated the distribution of the data obtained for the different enzymes across EC classes by normalizing each MD-derived descriptor to the corresponding experimental value (Figure 10). From the RMSD distribution (Figure 10A), it is evident that Finenzyme successfully generated the vast majority of enzyme variants that remain highly stable and properly folded during MD simulations, as indicated by ratio values close to 1.0 and, in most cases, lower than those observed for the corresponding crystallographic reference structures. The only notable exception is represented by protein 1ODE (EC 5.4.99.5), for which all generated sequences exhibit higher average RMSD values compared with the reference enzyme. However, it is important to note that the reference structure itself displays exceptional stability (RMSD = 1.06 Å), making it inherently challenging to obtain variants with numerically lower RMSD values. Consequently, although the generated sequences appear to deviate more markedly from the reference in the graphical representation, their average RMSD values remain below 2.0 Å, indicative of high structural stability. Consistently, 77.8% of these variants fell within the applied stability threshold (see Materials and methods and Table 5).

Similar trends were observed for the MD descriptors more directly related to conformational dynamics (i.e., RMSF, RoG, and SASA), which provide more insight into potential catalytic competence. In fact, the RMSF distribution (Figure 10B) showed that the vast majority of data points cluster around the ratio of 1.0 relative to the crystallographic reference structures. However, in contrast to the RMSD results, a slight skew toward higher values is evident, with fewer cases falling below the 1.0 reference value. As in the case of RMSD, 1ODE represents a notable outlier since, also in this case, the reference structure exhibited an exceptionally low RMSF value (0.73 Å), making it particularly challenging to obtain variants with reduced flexibility. Consequently, none of the generated sequences display RMSF values below the reference value. Nevertheless, only 9 out of 36 variants (25.0%) fell outside the applied threshold, indicating that most still retain acceptable dynamic properties. The RoG distribution (Figure 10C) showed values consistently close to the reference ratio of 1.0, with only 8 out of 263 sequences (3.0%) exceeding the defined threshold. Notably, in the case of protein 3RMI (EC 5.4.99.5), the vast majority of generated variants exhibited mean RoG values markedly lower than those of the crystallographic reference structure, suggesting a tendency toward increased compactness. This feature may be advantageous in the context of enzyme engineering, as enhanced structural compactness is often associated with improved stability under harsh industrial conditions, such as elevated temperature or extreme pH. A comparable trend is observed for the same protein in the SASA distribution (Figure 10D). In this plot, the data displayed a strong tendency to cluster around the reference value, although in this case the behavior appears more enzyme-dependent. Specifically, variants derived from 1LLU, 1DCT, and 6NDL predominantly populate the upper range of the distribution, whereas those associated with 1ODE and 1WNL are largely shifted toward lower values.

## 4 Conclusions and future developments

Accomplishing MD simulations on 236 Finenzyme-generated structures spanning four EC enzyme classes, each evolved over 250 ns (totaling 59 μs of simulation time), we confirmed that the designed sequences reliably adopt biologically plausible conformations. Across all classes, 74–95% of the models maintain stable tertiary structures and correct folding throughout the trajectories, with 195 out of 236 structures (82.6%) exhibiting sustained stability. Moreover, 163 out of 236 sequences (69.1%) exhibit structural features that may be compatible with a potential catalytic competence, according to our limited simulation-derived assessments. These percentages refer to the filtered candidate set and should not be read as the stability rate of all Finenzyme-generated sequences. A minority of candidates (roughly 5–26% across classes) failed the stability or potential catalytic competence criteria, typically through one of a few recurring modes observed in the trajectories: partial or full unfolding, mainly persistently elevated RMSF or SASA. Such outcomes are consistent with the stochastic nature of autoregressive sampling: not every draw from the learned distribution yields a stable sequence, which is precisely the motivation for the filtering step and may also reflect pLDDT mispredictions not captured by our threshold, loss of structural motifs during generation, or cofactor-binding-site incompatibilities. A systematic, quantitative failure analysis that could inform future fine-tuning iterations is left for follow-up work.

Remarkably, for sequences generated under reduced generative freedom (*p* = 0.5), we observed slightly higher proportions of stable and properly folded variants, as well as a larger fraction of sequences exhibiting conformational ensembles during MD simulations comparable to those of the reference crystal structures, relative to those obtained at *p* = 0.75. Specifically, 70/82 (85.4%) of the variants at *p* = 0.5 were classified as stable based on their average RMSD values, compared with 82/109 (75.2%) at *p* = 0.75. Similarly, 59/82 (71.9%) of the variants generated at *p* = 0.5 retained structural features consistent with potential catalytic competence, compared with 67/109 (61.5%) at *p* = 0.75, as inferred from average RMSF, RoG, and SASA values. It should be noted, however, that these MD metrics have inherent limitations and provide only a preliminary indication of catalytic competence. Taken together, these results indicate that Finenzyme effectively balances sequence diversity and structural reliability, with reduced generative freedom favoring the design of enzyme variants that more consistently preserve stability and potentially functionally relevant conformational properties.

Finally, this study demonstrates the efficacy of integrating artificial intelligence methodologies, such as Finenzyme, with MD simulations as a powerful strategy for enzyme engineering. Notably, several generated sequences exhibited greater predicted stability and residue diversity than their reference counterparts, enabling the design of mutant variants with potential enhanced catalytic activity or stability while preserving the reference fold. Such improvements could be leveraged in industrial applications to increase process efficiency or boost the yield of desired products. Furthermore, enzymes typically operate under mild conditions, near-ambient temperatures, neutral pH, and aqueous environments, thereby reducing energy consumption and eliminating the need for hazardous reagents or extreme conditions characteristic of conventional chemical syntheses. This translates into lower emissions and smaller carbon footprints for chemical processes. In essence, employing more efficient enzymes in the synthesis of organic compounds embodies the core principles of green chemistry, offering a sustainable and environmentally friendly alternative to traditional catalytic methods. In conclusion, our findings indicate that the generated sequences using Finenzyme exhibit structural stability and maintain features consistent with native architectures. However, they should be regarded as candidates for future experimental testing to determine their catalytic function.

Future developments include analysis of generated enzymes from different Protein Language Models (Both general ones and fine-tuned), moreover, we note that the 80% sequence-identity cut-off used in the novelty filter bounds redundancy but does not by itself establish structural novelty: sequences below this threshold can still belong to the same fold and family, as is the case here by design. Quantifying active-site novelty (for example, conservation and spatial arrangement of catalytic residues and motif diversity) is a planned direction for future work.

## Data Availability

Finenzyme code is available through GitHub: https://github.com/AnacletoLAB/Finenzyme. The authors will provide any additional files upon reasonable request, including PDB files of the generated sequences, MD input files and parameter sets, MD simulation trajectories, and any raw experimental and computational data.
