# Supplementary material for: Protein language model-generated enzyme sequences exhibit high stability in molecular dynamics simulations
Source: Front Artif Intell. 2026 Aug 31;9:1844189. doi: 10.3389/frai.2026.1844189 (PMC13572599; doi:10.3389/frai.2026.1844189)
Supplement: Supplementary file 1 [file Data_Sheet_1.PDF]

*Supplementary Materials for:*

# Protein Language Model-Generated Enzyme Sequences Exhibit High Stability in Molecular Dynamics Simulations

Enrico Mario Alessandro Fassi[1,*], Marco Nicolini[2], Emanuele Saitto[2], Giorgio Valentini[2,3], Elena Casiraghi[2,3], and Giovanni Grazioso[1]

[1]Dipartimento di Scienze Farmaceutiche, Università degli Studi di Milano, Italy, {enrico.fassi, giovanni.grazioso}@unimi.it

[2]AnacletoLab – Dipartimento di Informatica, Università degli Studi di Milano, Italy, {marco.nicolini, emanuele.saitto, elena.casiraghi, giorgio.valentini}@unimi.it

[3]ELLIS – European Laboratory for Learning and Intelligent Systems, Milan Unit, Italy

[*]corresponding author

## Molecular Dynamics details

To remove initial atom-atom clashes in the protein structure, the system was subjected to a multi-step relaxation protocol: (*i*) energy minimization with backbone atoms restrained by a harmonic potential of 20 $kcal \cdot mol^{-1} \cdot Å^{-2}$ for up to 10,000 steps, or until an energy gradient of 0.2 $kcal \cdot mol^{-1} \cdot Å^{-1}$ was reached; and (*ii*) subsequent energy minimization for up to 100,000 steps, or until an energy gradient of 0.0001 $kcal \cdot mol^{-1} \cdot Å^{-1}$ was attained, without any positional restraints. The system was then gradually heated to 300 K over 60 ps, while the pressure was raised to 1 atm. During the MD simulations, temperature and pressure were maintained constant using a Langevin thermostat and a Monte Carlo barostat, respectively [1,2]. Bonds involving hydrogen atoms were constrained using the SHAKE algorithm. All calculations were performed applying a cutoff of 9.0 Å to non-bonded van der Waals interactions, while electrostatic interactions were treated with the Particle Mesh Ewald (PME) method [3].

[1] Loncharich RJ, Brooks BR, Pastor RW. Langevin dynamics of peptides: the frictional dependence of isomerization rates of N-acetylalanyl-N'-methylamide. Biopolymers. 1992;32(5):523-35. doi: 10.1002/bip.360320508.

[2] Åqvist J, Wennerström P, Nervall M, Bjelic S, Brandsdal BO. Molecular dynamics simulations of water and biomolecules with a Monte Carlo constant pressure algorithm. Chem. Phys. Lett. 2004;384:288-294. doi: 10.1016/j.cplett.2003.12.039.

[3] Harris JA, Liu R, Martins de Oliveira V, Vázquez-Montelongo EA, Henderson JA, Shen J. GPU-Accelerated All-Atom Particle-Mesh Ewald Continuous Constant pH Molecular Dynamics in Amber. J Chem Theory Comput. 2022;18(12):7510-7527. doi: 10.1021/acs.jctc.2c00586.

**Table S1.** Mean reference values (μ_ref), standard deviations (σ), and applied thresholds for RMSD, RMSF, RoG, and SASA calculated for each subset of Finenzyme-generated sequences analyzed by MD simulations, using the crystal structures of 1LLU, 1DCT, 3RMI, 1ODE, 1WNL, and 6NDL as references. Threshold values were defined as μ_ref + σ for RMSD and RMSF, and as μ_ref + 2σ for RoG and SASA. An exception was made for protein subsets referencing 1ODE, for which the thresholds for RMSD and RMSF were also defined as μ_ref + 2σ.

| Structure | Descriptor | RMSD | RMSF | RoG | SASA |
|---|---|---|---|---|---|
| **1LLU (EC 1.1.1.1)** | μ_ref | 2.89 Å | 1.20 Å | 20.7 Å | 15,538 Å$^2$ |
| | σ | 0.66 | 0.19 | 0.45 | 786 |
| | threshold | 3.55 Å | 1.39 Å | 21.6 Å | 17,110 Å$^2$ |
| **1DCT (EC 2.1.1.37)** | μ_ref | 2.55 Å | 0.79 Å | 20.1 Å | 13,527 Å$^2$ |
| | σ | 0.50 | 0.30 | 0.43 | 709 |
| | threshold | 3.05 Å | 1.09 Å | 21.0 Å | 14,946 Å$^2$ |
| **3RMI (EC 5.4.99.5)** | μ_ref | 6.06 Å | 2.85 Å | 19.1 Å | 8,323 Å$^2$ |
| | σ | 1.88 | 1.02 | 1.78 | 592 |
| | threshold | 7.94 Å | 3.87 Å | 22.7 Å | 9,508 Å$^2$ |
| **1ODE (EC 5.4.99.5)** | μ_ref | 1.06 Å | 0.73 Å | 13.7 Å | 7,018 Å$^2$ |
| | σ | 0.48 | 0.22 | 0.18 | 168 |
| | threshold | 2.02 Å | 1.17 Å | 14.1 Å | 7,354 Å$^2$ |
| **1WNL (EC 6.3.4.15)** | μ_ref | 1.75 Å | 0.95 Å | 17.2 Å | 11,289 Å$^2$ |
| | σ | 0.59 | 0.15 | 0.33 | 433 |
| | threshold | 2.34 Å | 1.10 Å | 17.9 Å | 12,154 Å$^2$ |
| **6NDL (EC 6.3.4.15)** | μ_ref | 3.33 Å | 1.42 Å | 21.9 Å | 15,376 Å$^2$ |
| | σ | 1.57 | 0.71 | 0.43 | 468 |
| | threshold | 4.90 Å | 2.13 Å | 22.8 Å | 16,312 Å$^2$ |

## EC 1.1.1.1 (alcohol dehydrogenase)

**Table S2.** Average RMSD (Å), RMSF (Å), RoG (Å), and SASA (Å²) values for the generated EC 1.1.1.1 enzymes at $p = 0.75$, compared to the reference structure 1LLU. Values exceeding the thresholds are highlighted in red.

| Structure | RMSD | RMSF | RoG | SASA | Structure | RMSD | RMSF | RoG | SASA |
|---|---|---|---|---|---|---|---|---|---|
| **1LLU** | **2.89** | **1.20** | **20.7** | **15538** | **418** | 2.82 | 1.33 | 20.1 | 15768 |
| **5** | 2.31 | 1.00 | 19.7 | 15421 | **423** | 2.43 | 1.12 | 20.4 | 15862 |
| **7** | 1.31 | 0.75 | 19.7 | 14917 | **440** | 2.54 | 0.89 | 20.3 | 15273 |
| **16** | 2.86 | 1.30 | 20.3 | 15655 | **450** | 2.06 | 1.12 | 20.7 | 16363 |
| **25** | 2.61 | 1.32 | 20.2 | 14949 | **455** | 3.99 | 1.64 | 20.9 | 17539 |
| **53** | 2.72 | 1.11 | 20.6 | 16025 | **496** | 2.81 | 1.10 | 20.1 | 16154 |
| **65** | 2.76 | 1.40 | 21.0 | 16911 | **502** | 2.02 | 1.18 | 20.8 | 16122 |
| **90** | 2.29 | 1.10 | 20.2 | 15803 | **513** | 3.16 | 1.18 | 21.2 | 17213 |
| **168** | 2.60 | 1.16 | 20.7 | 15645 | **524** | 2.37 | 1.09 | 20.7 | 16036 |
| **190** | 2.01 | 1.22 | 20.0 | 15340 | **527** | 1.61 | 0.98 | 19.9 | 14145 |
| **211** | 3.59 | 1.23 | 20.9 | 17456 | **573** | 3.03 | 1.22 | 21.3 | 16043 |
| **230** | 2.28 | 1.24 | 20.0 | 15749 | **575** | 2.81 | 1.26 | 20.7 | 15338 |
| **239** | 1.67 | 0.92 | 20.4 | 15414 | **598** | 3.25 | 1.31 | 21.0 | 17451 |
| **241** | 2.45 | 1.21 | 21.3 | 16732 | **616** | 3.19 | 1.46 | 20.5 | 16078 |
| **252** | 2.51 | 1.25 | 20.7 | 16471 | **663** | 2.50 | 1.26 | 21.0 | 16155 |
| **260** | 2.31 | 1.38 | 20.4 | 16295 | **680** | 2.99 | 1.22 | 20.9 | 16879 |
| **281** | 2.37 | 1.13 | 20.8 | 16422 | **717** | 3.10 | 1.36 | 20.7 | 16208 |
| **286** | 3.64 | 1.44 | 21.2 | 17245 | **749** | 2.00 | 1.14 | 20.6 | 15680 |
| **364** | 2.46 | 1.07 | 20.3 | 15131 | **758** | 2.74 | 1.05 | 19.7 | 14422 |
| **389** | 2.89 | 1.15 | 21.2 | 15308 | **796** | 1.89 | 0.96 | 20.1 | 15219 |
| **391** | 3.14 | 1.48 | 20.7 | 16807 | **837** | 2.50 | 1.29 | 20.9 | 15719 |
| **398** | 2.90 | 1.29 | 20.4 | 15687 | **910** | 5.20 | 1.82 | 21.3 | 16244 |
| **416** | 2.06 | 1.07 | 20.5 | 14843 | **991** | 2.00 | 1.12 | 19.9 | 15352 |

## EC 2.1.1.37 (DNA(cytosine-5)-methyltransferase)

**Table S3.** Average RMSD (Å), RMSF (Å), RoG (Å), and SASA (Å²) values for the generated EC 2.1.1.37 enzymes at $p = 0.5$ (top) and $p = 0.75$ (bottom), compared to the reference structure 1DCT. Values exceeding the respective thresholds are highlighted in red.

| Structure | RMSD | RMSF | RoG | SASA | Structure | RMSD | RMSF | RoG | SASA |
|---|---|---|---|---|---|---|---|---|---|
| **1DCT** | **2.55** | **0.79** | **20.1** | **13527** | **472** | 1.70 | 1.07 | 20.1 | 13334 |
| **17** | 1.63 | 0.93 | 20.3 | 13747 | **613** | 1.65 | 0.83 | 20.2 | 13222 |
| **64** | 1.87 | 0.88 | 20.2 | 13515 | **731** | 2.00 | 0.88 | 20.3 | 13550 |
| **82** | 2.28 | 1.74 | 20.3 | 13787 | **870** | 1.83 | 1.60 | 20.4 | 14034 |
| **152** | 2.03 | 1.57 | 20.3 | 13734 | **900** | 1.90 | 0.89 | 20.3 | 13352 |
| **250** | 1.82 | 0.95 | 20.3 | 13942 | **996** | 1.93 | 1.58 | 20.2 | 13774 |
| **1DCT** | **2.55** | **0.79** | **20.1** | **13527** | **600** | 2.23 | 1.32 | 20.3 | 14573 |
| **33** | 2.25 | 0.97 | 20.4 | 13930 | **602** | 1.87 | 0.96 | 20.7 | 14824 |
| **124** | 1.80 | 0.82 | 20.5 | 14186 | **605** | 2.67 | 1.24 | 20.9 | 15258 |
| **321** | 2.69 | 0.92 | 20.0 | 13156 | **807** | 1.87 | 1.33 | 20.3 | 14516 |
| **381** | 2.26 | 1.44 | 20.2 | 14334 | **841** | 2.03 | 0.92 | 20.6 | 15114 |
| **442** | 2.04 | 1.14 | 20.5 | 14153 | **966** | 3.87 | 1.59 | 22.2 | 16001 |
| **531** | 2.86 | 0.98 | 20.6 | 14648 | | | | | |

## EC 5.4.99.5 (chorismate mutase)

**Table S4.** Average RMSD (Å), RMSF (Å), RoG (Å), and SASA ($\text{Å}^2$) values for the generated EC 5.4.99.5 enzymes at $p$ = 0.5 (top) and $p$ = 0.75 (bottom), compared to reference structure 3RMI. Values exceeding the respective thresholds are highlighted in red.

| Structure | RMSD | RMSF | RoG | SASA | Structure | RMSD | RMSF | RoG | SASA |
|---|---|---|---|---|---|---|---|---|---|
| **3RMI** | **6.06** | **2.85** | **19.1** | **8323** | **341** | 6.44 | 2.75 | 18.8 | 8321 |
| **22** | 5.08 | 2.37 | 16.8 | 7322 | **411** | 8.22 | 3.45 | 17.2 | 7593 |
| **45** | 11.36 | 6.36 | 19.3 | 8253 | **488** | 7.33 | 3.94 | 18.1 | 7931 |
| **106** | 6.64 | 2.16 | 16.8 | 7286 | **532** | 12.42 | 4.18 | 15.4 | 7584 |
| **136** | 9.01 | 2.47 | 21.1 | 8784 | **563** | 7.06 | 4.19 | 19.9 | 8636 |
| **215** | 5.74 | 2.93 | 18.1 | 7791 | **661** | 11.17 | 4.41 | 16.1 | 8019 |
| **221** | 5.76 | 3.01 | 17.8 | 8080 | **715** | 5.48 | 2.43 | 19.6 | 8077 |
| **227** | 4.52 | 1.55 | 15.9 | 7531 | **833** | 7.96 | 4.74 | 20.3 | 8624 |
| **262** | 7.10 | 3.56 | 18.6 | 8048 | | | | | |
| **3RMI** | **6.06** | **2.85** | **19.1** | **8323** | **642** | 6.14 | 2.75 | 14.8 | 7351 |
| **12** | 10.8 | 3.47 | 16.5 | 8269 | **659** | 7.10 | 2.26 | 18.1 | 7833 |
| **92** | 6.73 | 2.51 | 15.0 | 7336 | **680** | 4.29 | 1.70 | 16.1 | 7477 |
| **137** | 7.19 | 2.84 | 15.4 | 7605 | **690** | 5.17 | 2.66 | 15.6 | 6845 |
| **178** | 8.14 | 3.37 | 15.0 | 7392 | **736** | 6.96 | 3.16 | 16.1 | 6996 |
| **210** | 5.82 | 1.93 | 16.2 | 7433 | **774** | 6.37 | 2.04 | 17.2 | 7890 |
| **337** | 7.64 | 3.06 | 17.7 | 8845 | **801** | 6.35 | 4.70 | 18.7 | 8148 |
| **453** | 8.28 | 4.87 | 17.6 | 8243 | **823** | 7.88 | 2.08 | 16.2 | 7067 |
| **513** | 5.44 | 3.41 | 16.6 | 6996 | **878** | 7.90 | 2.89 | 17.2 | 8039 |
| **519** | 6.17 | 2.57 | 15.8 | 6838 | **892** | 6.01 | 2.74 | 21.4 | 8965 |
| **577** | 5.80 | 2.01 | 16.1 | 7265 | **893** | 5.04 | 2.47 | 14.4 | 6753 |

**Table S5.** Average RMSD (Å), RMSF (Å), RoG (Å), and SASA ($\text{Å}^2$) values for the generated EC 5.4.99.5 enzymes at $p$ = 0.5 (top) and $p$ = 0.75 (bottom), compared to reference structure 1ODE. Values exceeding the respective thresholds are highlighted in red.

| Structure | RMSD | RMSF | RoG | SASA | Structure | RMSD | RMSF | RoG | SASA |
|---|---|---|---|---|---|---|---|---|---|
| **1ODE** | **1.06** | **0.73** | **13.7** | **7018** | **805** | 1.59 | 1.11 | 13.8 | 7010 |
| **64** | 1.93 | 1.26 | 13.9 | 7039 | **877** | 1.46 | 0.95 | 13.8 | 6803 |
| **546** | 1.60 | 1.08 | 14.1 | 7071 | **885** | 1.61 | 0.99 | 14.0 | 6847 |
| **790** | 1.62 | 1.05 | 14.0 | 7029 | | | | | |
| **1ODE** | **1.06** | **0.73** | **13.7** | **7018** | **448** | 1.82 | 1.13 | 13.8 | 6827 |
| **8** | 1.79 | 0.99 | 13.7 | 6929 | **483** | 2.00 | 1.12 | 13.9 | 6689 |
| **17** | 2.09 | 1.87 | 13.8 | 6866 | **492** | 1.40 | 1.13 | 13.8 | 6831 |
| **73** | 1.49 | 1.27 | 13.8 | 6862 | **551** | 1.54 | 1.20 | 13.9 | 6954 |
| **101** | 1.22 | 1.06 | 13.7 | 6674 | **557** | 3.26 | 1.26 | 13.9 | 7356 |
| **146** | 1.57 | 0.89 | 13.7 | 6873 | **589** | 1.24 | 1.03 | 13.7 | 6753 |
| **158** | 1.14 | 0.85 | 13.8 | 7054 | **591** | 1.99 | 1.20 | 13.7 | 6781 |
| **201** | 1.84 | 1.09 | 13.7 | 7020 | **596** | 2.61 | 1.02 | 13.5 | 6925 |
| **220** | 1.61 | 0.88 | 13.7 | 6823 | **626** | 2.31 | 1.13 | 13.6 | 6886 |
| **234** | 1.51 | 1.23 | 13.8 | 6914 | **631** | 1.51 | 0.90 | 13.7 | 6883 |
| **258** | 1.85 | 1.08 | 14.0 | 6901 | **685** | 1.72 | 0.93 | 13.7 | 6667 |
| **261** | 3.02 | 1.24 | 13.7 | 7048 | **742** | 1.89 | 0.92 | 13.6 | 6592 |
| **285** | 1.57 | 1.47 | 13.8 | 6552 | **747** | 2.22 | 0.89 | 13.7 | 6753 |
| **320** | 1.26 | 0.92 | 13.8 | 6851 | **779** | 2.11 | 1.25 | 13.9 | 7022 |
| **352** | 1.92 | 1.55 | 14.5 | 6630 | **793** | 2.49 | 0.87 | 13.5 | 6986 |
| **365** | 1.59 | 0.87 | 14.0 | 6545 | | | | | |

## EC 6.3.4.15 (biotin-protein ligase)

**Table S6.** Average RMSD (Å), RMSF (Å), RoG (Å), and SASA (Å²) values for the generated EC 6.3.4.15 enzymes at $p$ = 0.5 (top) and $p$ = 0.75 (bottom), compared to reference structure 1WNL. Values exceeding the respective thresholds are highlighted in red.

| Structure | RMSD | RMSF | RoG | SASA | Structure | RMSD | RMSF | RoG | SASA |
|---|---|---|---|---|---|---|---|---|---|
| **1WNL** | **1.75** | **0.95** | **17.2** | **11289** | **610** | 2.12 | 0.94 | 17.2 | 10914 |
| **126** | 1.71 | 0.95 | 17.6 | 10976 | **643** | 2.86 | 1.22 | 17.8 | 11225 |
| **142** | 1.93 | 0.97 | 17.2 | 10790 | **648** | 2.13 | 1.08 | 17.4 | 10769 |
| **183** | 2.26 | 0.88 | 17.4 | 10600 | **682** | 1.80 | 0.94 | 17.9 | 10655 |
| **230** | 1.91 | 0.91 | 17.0 | 10939 | **700** | 1.73 | 0.94 | 17.1 | 10869 |
| **231** | 1.75 | 0.93 | 17.7 | 11241 | **749** | 2.64 | 1.24 | 17.1 | 11020 |
| **264** | 2.79 | 1.33 | 17.6 | 11912 | **765** | 1.80 | 0.96 | 17.2 | 10859 |
| **368** | 1.73 | 0.96 | 17.3 | 11275 | **780** | 1.88 | 0.96 | 17.6 | 10754 |
| **375** | 1.46 | 0.87 | 17.9 | 10739 | **837** | 1.85 | 0.90 | 17.9 | 10605 |
| **401** | 1.60 | 0.97 | 17.4 | 11116 | **871** | 2.95 | 1.20 | 17.3 | 11688 |
| **444** | 2.07 | 1.07 | 17.0 | 11384 | **915** | 1.73 | 0.94 | 17.4 | 10558 |
| **473** | 1.48 | 0.82 | 17.8 | 10675 | **937** | 2.62 | 0.98 | 17.3 | 10978 |
| **479** | 1.92 | 1.17 | 17.5 | 11376 | **947** | 1.84 | 0.88 | 17.9 | 10905 |
| **576** | 1.79 | 0.96 | 17.9 | 11048 | **995** | 1.74 | 0.94 | 17.9 | 10713 |
| **577** | 1.48 | 0.83 | 17.8 | 10914 | | | | | |
| **1WNL** | **1.75** | **0.95** | **17.2** | **11289** | **399** | 1.93 | 1.17 | 17.3 | 11692 |
| **48** | 3.39 | 1.24 | 17.3 | 11749 | **401** | 4.67 | 1.37 | 17.6 | 11313 |
| **81** | 1.65 | 0.93 | 17.2 | 11094 | **403** | 2.32 | 1.07 | 17.3 | 11191 |
| **99** | 1.63 | 0.99 | 17.4 | 11161 | **411** | 1.77 | 1.18 | 17.3 | 11178 |
| **121** | 2.64 | 1.24 | 17.5 | 12432 | **417** | 2.84 | 1.39 | 17.5 | 12342 |
| **139** | 2.01 | 1.13 | 17.3 | 11010 | **424** | 2.22 | 0.98 | 17.0 | 10671 |
| **146** | 1.95 | 1.05 | 17.2 | 10903 | **450** | 2.04 | 1.07 | 17.1 | 10673 |
| **156** | 2.36 | 1.29 | 17.9 | 11995 | **464** | 3.04 | 1.30 | 17.3 | 11443 |
| **198** | 1.99 | 1.13 | 17.4 | 11593 | **495** | 1.77 | 0.86 | 17.7 | 11715 |
| **245** | 1.81 | 1.05 | 17.4 | 11280 | **497** | 2.11 | 0.95 | 17.4 | 10889 |
| **279** | 1.85 | 1.08 | 17.2 | 11015 | **587** | 2.09 | 0.99 | 17.8 | 11165 |
| **308** | 1.76 | 1.08 | 17.3 | 10846 | **658** | 2.43 | 1.25 | 17.3 | 11848 |
| **323** | 2.52 | 1.26 | 17.5 | 11307 | **671** | 2.14 | 1.03 | 17.4 | 10892 |
| **354** | 1.80 | 1.02 | 17.3 | 11137 | **686** | 1.84 | 1.19 | 17.8 | 11700 |
| **365** | 2.51 | 1.30 | 17.6 | 11308 | **689** | 3.92 | 1.43 | 17.9 | 11819 |
| **376** | 2.06 | 1.06 | 17.2 | 10983 | | | | | |

**Table S7.** Average RMSD (Å), RMSF (Å), RoG (Å), and SASA (Å²) values for the generated EC 6.3.4.15 enzymes at $p$ = 0.5 (top) and $p$ = 0.75 (bottom), compared to reference structure 6NDL. Values exceeding the respective thresholds are highlighted in red.

| Structure | RMSD | RMSF | RoG | SASA | Structure | RMSD | RMSF | RoG | SASA |
|---|---|---|---|---|---|---|---|---|---|
| **6NDL** | **3.33** | **1.42** | **21.9** | **15376** | **424** | 2.07 | 1.33 | 22.1 | 15754 |
| **37** | 3.63 | 2.13 | 23.0 | 16654 | **453** | 6.24 | 2.81 | 22.9 | 16317 |
| **42** | 2.30 | 1.23 | 22.5 | 15895 | **608** | 2.54 | 1.48 | 21.1 | 15200 |
| **143** | 2.40 | 1.23 | 22.2 | 15329 | **612** | 2.37 | 1.28 | 22.3 | 15947 |
| **246** | 3.15 | 1.80 | 22.6 | 15612 | **616** | 2.33 | 1.30 | 22.2 | 16429 |
| **337** | 2.58 | 1.31 | 22.5 | 15988 | **647** | 2.31 | 1.26 | 22.2 | 16115 |
| **363** | 2.92 | 1.57 | 22.5 | 16464 | **847** | 2.91 | 2.06 | 21.9 | 15920 |
| **390** | 2.43 | 1.20 | 22.2 | 15323 | **868** | 2.20 | 1.20 | 22.3 | 16070 |
| **394** | 4.45 | 1.67 | 22.4 | 15962 | **877** | 2.35 | 1.16 | 22.1 | 16553 |
| **398** | 2.25 | 1.04 | 22.0 | 15212 | **933** | 2.43 | 1.21 | 22.3 | 15655 |
| **414** | 2.29 | 1.18 | 22.2 | 15598 | **950** | 2.37 | 1.41 | 22.3 | 15665 |

| | | | | | | | | | |
|---|---|---|---|---|---|---|---|---|---|
| **6NDL** | **3.33** | **1.42** | **21.9** | **15376** | **705** | 2.19 | 1.22 | 21.2 | 16255 |
| **190** | 3.26 | 2.11 | 22.1 | 15355 | **709** | 5.52 | 3.13 | 22.7 | 16372 |
| **252** | 4.33 | 1.45 | 21.9 | 15600 | **822** | 2.94 | 1.21 | 21.9 | 15221 |
| **506** | 4.13 | 1.31 | 21.7 | 15448 | **830** | 2.88 | 1.71 | 22.7 | 16481 |
| **589** | 2.44 | 1.61 | 22.1 | 15967 | **850** | 8.84 | 3.38 | 22.0 | 15443 |
| **595** | 5.75 | 1.97 | 22.3 | 16488 | **859** | 2.69 | 1.87 | 22.5 | 15853 |
| **674** | 2.74 | 1.48 | 22.1 | 15914 | **906** | 5.63 | 2.21 | 23.0 | 16676 |
| **682** | 2.45 | 1.31 | 21.6 | 16338 | **930** | 3.44 | 1.62 | 22.5 | 16431 |
| **696** | 7.15 | 4.47 | 22.9 | 16751 | | | | | |
